# The role of tele‐ultrasound for real‐time remote guidance for femoral nerve blocks in the emergency department

**DOI:** 10.1002/aet2.11053

**Published:** 2025-02-14

**Authors:** Simanjit K. Mand, Jessica Koehler, Jessica R. Baez, Patrick G. Minges, Lori A. Stolz

**Affiliations:** ^1^ University of Cincinnati College of Medicine Cincinnati Ohio USA; ^2^ University of Wisconsin School of Medicine and Public Health Madison Wisconsin USA; ^3^ University of Michigan Medical School Ann Arbor Michigan USA

## Abstract

The use of point‐of‐care ultrasound (POCUS) is integral to the practice of emergency medicine, particularly for procedural guidance. While simulated repetitions can aid in educating physicians in rare or difficult procedures, they cannot replace the need for real‐time supervision and guidance especially when a learner is performing a procedure on a patient for the first time. However, procedural experts may not be immediately available in many clinical environments. We present a framework for a tele‐ultrasound protocol for use with femoral nerve blocks in which expert proceduralists can be readily available to assist with ultrasound‐guided procedures with ease and accessibility.

## BACKGROUND

Tele‐ultrasound, the guiding and providing of feedback on transmitted sonographic images in real time, is a form of telemedicine that can be utilized in any training or clinical environment when skilled educators may be limited or not physically present.^1^ During the early phases of the COVID‐19 pandemic, many residency programs pivoted to virtual didactics; however, this format is suboptimal for procedural training. While simulation and dedicated practice are crucial, they cannot replace the supervision and guidance required for a learner's procedural attempt on a real patient.

Several studies have demonstrated the feasibility of expert emergency physicians using tele‐ultrasound to help novice users learn scanning techniques and identify pathology with point‐of‐care‐ultrasound use in remote settings.^1^, ^2^, ^3^, ^4^, ^5^ However, there is only preliminary literature demonstrating tele‐ultrasound use in emergency medicine for hands‐on procedural education.^6^, ^7^

## EXPLANATION

A multidisciplinary group (including emergency medicine, orthopedic surgery, and trauma surgery) developed a protocol for our hospital system involving a single injection femoral nerve block performed at the time of presentation for adults with isolated femur fractures. Exclusion criteria included patients who declined or were unable to provide consent, had an allergy to local anesthetic, presented with a crush injury, and a lack of provider privileging for the procedure (preliminary procedural training via didactics and simulation required for privileging).

To provide the quantity and quality of educational oversight for this endeavor, we identified a need for remote, real‐time tele‐ultrasound supervision to assist with implementation. The tele‐ultrasound software provides real‐time video and audio stream of the provider's face and/or hands in addition to the real‐time transmission of the sonographic video to help guide patient positioning, landmark identification, troubleshooting of needle trajectory, and successful anesthetic injection. The proctor can guide the learner through the procedure, make notations on the ultrasound image, and provide real‐time verbal guidance and education. The tele‐ultrasound protocol (Table 1) was finalized via iterative revisions made in response to simulated runs conducted by ultrasound fellows prior to clinical implementation.

**TABLE 1:** Tele‐ultrasound procedural steps from provider and proctor points of view.

Provider point of view		Proctor point of view
Step 1: Activate tele‐ultrasound software^a^	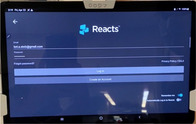	
Step 2: Access proctor call list	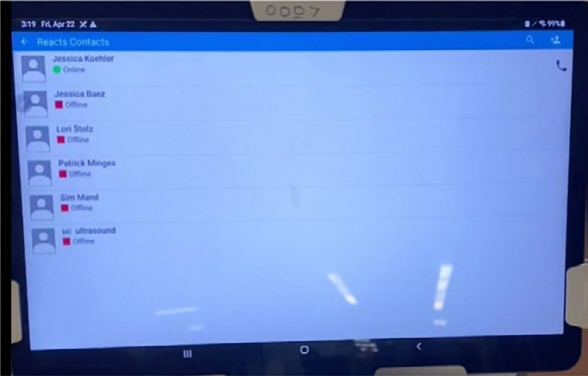	
Step 3: Call proctor	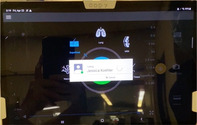	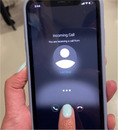
Step 4: Use real‐time video and audio stream of provider face/hands, proctor face, and real‐time transmission of sonographic video	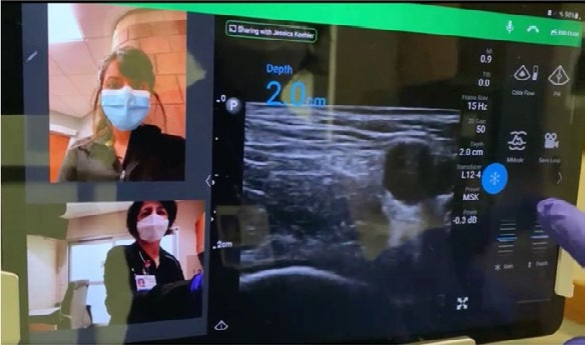	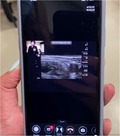
Step 5: Proctor can use pointer to provide notations on provider's screen for more targeted guidance	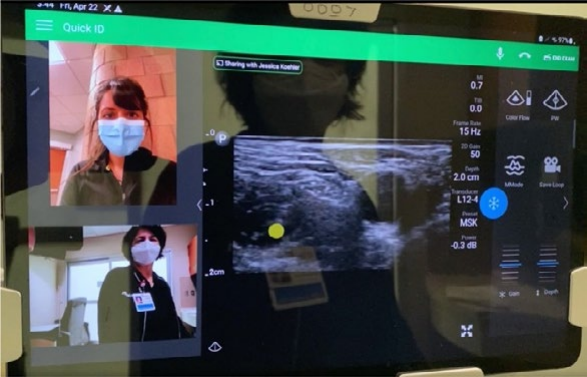	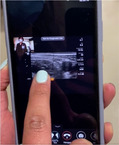

^a^ Reacts software (Philips Ultrasound) demonstrated here.

The emergency department (ED) provider (resident or faculty physician) identified a patient meeting criteria for a femoral nerve block. The provider then obtained written patient consent, prepared for the procedure, activated the tele‐ultrasound software (Reacts, Philips Ultrasound), and called the proctor. All ultrasound‐trained faculty and fellows with femoral nerve block experience and training were chosen as proctors. The proctor received the call via mobile phone application anywhere off campus with WiFi access and watched, discussed, and guided the procedure as it was performed.

## DESCRIPTION

Our providers performed 2 femoral nerve blocks in the 6 months prior to protocol introduction compared with 31 blocks in the 6 months after. No major or minor adverse events have been reported to date.

The program evaluation, completed via an anonymous survey, was exempt from the institutional review board. The survey was distributed to emergency physician providers who were actively working at the academic ED site at the time of the protocol implementation. The survey included a mix of Likert‐style questions and additional open‐ended questions to gather qualitative data (see Appendix A).

A total of 27 provider responses were received (7 attending and 20 resident physicians). Only 9% of providers had performed any femoral nerve blocks before the protocol, while 86% performed at least one after tele‐ultrasound introduction. Ninety percent reported that tele‐ultrasound contributed to procedural success, and 90% reported that it contributed to better patient care. A total of 62% reported it was somewhat/very easy to use with 76% reporting they would be somewhat/extremely likely to use tele‐ultrasound guidance again for learning a new or unfamiliar procedure.

Providers reported that the main areas of strength for the tele‐ultrasound protocol were that it ensured availability of a procedural expert for real‐time guidance and provided providers with reassurance and an opportunity for easier learning. The most commonly identified area for improvement was technical concerns, with some reporting WiFi and software connectivity as an issue. One provider also noted difficulty ensuring the ultrasound machine with the appropriate software access was readily available when needed. Our successful use of tele‐ultrasound for procedural guidance can be replicated in other EDs and, more broadly, in remote practice areas that may benefit from real‐time expert guidance for novel or rare procedures.

## AUTHOR CONTRIBUTIONS

Study concept and design: Simanjit K. Mand, Jessica Koehler, Jessica R. Baez, Patrick G. Minges, Lori A. Stolz. Acquisition of data: Simanjit K. Mand, Lori A. Stolz. Analysis and interpretation of the data: Simanjit K. Mand, Lori A. Stolz. Drafting of the manuscript: Simanjit K. Mand, Jessica Koehler, Lori A. Stolz. Critical revision of the manuscript for important intellectual content: Simanjit K. Mand, Jessica Koehler, Jessica R. Baez, Patrick G. Minges, Lori A. Stolz. Statistical expertise: NA. Obtained funding: NA. Administrative, technical, or material support: NA. Study supervision: NA.

## CONFLICT OF INTEREST STATEMENT

Dr. Stolz has received fees for consulting from ThinkSono, Butterfly Ultrasound, Caption Health and Philips Ultrasound. The other authors declare no conflicts of interest.
